# Transitions of foliar mycobiota community and transcriptome in response to pathogenic conifer needle interactions

**DOI:** 10.1038/s41598-022-11907-0

**Published:** 2022-05-12

**Authors:** Jessa P. Ata, Jorge R. Ibarra Caballero, Zaid Abdo, Stephen J. Mondo, Jane E. Stewart

**Affiliations:** 1 Department of Agricultural Biology, Colorado State University, Fort Collins, CO USA; 2 Department of Forest Biological Sciences, University of the Philippines Los Baños, Laguna, Philippines; 3 Department of Microbiology, Immunology and Pathology, Colorado State University, Fort Collins, CO USA; 4 Department of Energy Joint Genome Institute, Lawrence Berkeley National Laboratory, Berkeley, CA USA

**Keywords:** Environmental microbiology, Fungi, Fungal pathogenesis

## Abstract

Profiling the host–mycobiota interactions in healthy vs. diseased forest ecosystems helps understand the dynamics of understudied yet increasingly important threats to forest health that are emerging due to climate change. We analyzed the structural and functional changes of the mycobiota and the responses of *Pinus contorta* in the Lophodermella needle cast pathosystem through metabarcoding and metatranscriptomics. When needles transitioned from asymptomatic to symptomatic, dysbiosis of the mycobiota occurred, but with an enrichment of *Lophodermella* pathogens. Many pathogenicity-related genes were highly expressed by the mycobiota at the necrotrophic phase, showing an active pathogen response that are absent in asymptomatic needles. This study also revealed that *Lophodermella* spp. are members of a healthy needle mycobiota that have latent lifestyles suggesting that other pine needle pathogens may have similar biology. Interestingly, *Pinus contorta* upregulated defense genes in healthy needles, indicating response to fungal recognition, while a variety of biotic and abiotic stresses genes were activated in diseased needles. Further investigation to elucidate the possible antagonistic interplay of other biotic members leading to disease progression and/or suppression is warranted. This study provides insights into microbial interactions in non-model pathosystems and contributes to the development of new forest management strategies against emerging latent pathogens.

## Introduction

Endophytes are microorganisms that colonize plant tissues without causing symptoms and are known to play a vital role in plant health^1^. The wide species diversity of endophytes has been associated with a suite of diverse, but often unknown or poorly understood, ecological functions^2^. In many host-endophyte interactions, their symbiosis with host plants provides beneficial effects on host plant fitness and survival amid stressors, particularly pests and pathogens^3^. Directly or indirectly, endophytes protect plants against pests and pathogens through improving plant physiology, hyperparasitism, production of secondary metabolites, etc.^4^. On the other hand, endophytes can also be harmful latent pathogens that remain dormant until favorable environmental conditions occur or when hosts become weakened when under stress^5^. Some foliar fungal endophytes can also increase disease severity by enabling pathogen infections^6,7^. These relationships, however, are only part of a complex continuum of host-endophyte interactions that have a significant impact on forest ecosystem health.

These diverse ecological roles of the microbiota can trigger different plant responses to microbial infection and invasion. While similar initial defense responses could be elicited by both pathogenic and non-pathogenic endophytes, some symbiotic microbes have sophisticated systems recognized by the host plant which result in the downregulation of defense genes^8^. Similarly, in a direct fungal-host interaction, Ref.^9^ demonstrated that unlike for endophytes, plants recognize pathogens via overproduction of host defense enzymes. These differing host responses by the host plant to pathogenic and endophytic fungal infections could likely indicate plant evolutionary adaptations that emerged through pathogenic interactions^10^.

Profiling the mycobiota in a pathosystem and their interaction with the host plant can improve our understanding of disease development and suppression. Shifts in the microbial diversity^11,12^ and gene expression of the microbiome and host in healthy versus diseased plants were demonstrated to have important structural and functional impacts on disease development^13,14^. This is particularly relevant as our view of disease development shifts from the classical “one-microbe-one disease” to a more complex nature that involves coinfection of a concert of microbial organisms interacting with their environment, or a pathobiome^11,15,16^. Conversely, disease suppressive activities of endophytic microbial consortia have been continuously explored to reduce disease impacts^17^.

*Lophodermella concolor* (Dearn.) Darker and *L. montivaga* Petrak of Rhytismataceae are potentially obligate pathogens causing needle cast on *Pinus contorta*^18^, which is naturally distributed along the western region of northern America. Disease symptoms on infected hosts include needle discoloration and defoliation which could negatively impact growth when severe^18^. Recently, two epidemics caused by these two pathogens were recorded in Colorado, USA^19^. Though found on the same host and in the same sites in Colorado, their ecological interaction on an individual host has not been well-documented. It has been reported that, among the infected sites, all but one had only a single pathogen occurrence based on development of hysterothecia or sexual fruiting body, which may be due to unknown ecological differences between the two species^19^ or ecological competition prevailed by the most dominant and/or aggressive pathogen^20^. Additionally, little information is known about the interaction between these pathogens and other fungal endophytes in *P. contorta*. However, invasion of other fungi was reported to inhibit ascocarp development^21^.

This study aims to understand the interaction between the needle mycobiota with *L. concolor* and with *L. montivaga* in *P. contorta*. Using next generation sequencing, we specifically examined the fungal endophytic community composition and gene expression in asymptomatic and symptomatic needles of *P. contorta* trees infected with *Lophodermella* pathogens. We also explored the differences in plant responses at asymptomatic and symptomatic states. We expected a unique fungal assemblage in asymptomatic needles that suppresses disease development compared to symptomatic needles. In symptomatic needles, the pathogenicity-related mechanisms of *L. concolor* or *L. montivaga* provide a competitive advantage either through niche competition or antagonism over other fungal endophytes lowering fungal diversity. Based on previously observed morphological and ecological differences between the two pathogens, we further hypothesized that variations exist in the gene expression profiles between *L. concolor* and *L. montivaga* and in their interaction within the host. Our findings partially confirm these hypotheses, and elucidate the interaction among the needle mycobiome and plant host in the transition from healthy to diseased state.

## Results

### Mycobiome sequences, composition and diversity

A total of 7,670,091 ITS contigs were obtained from the DNA of 60 symptomatic and 58 asymptomatic needle samples obtained after screening and filtering. Of these, 705,680 were unique contigs which were then further reduced to 664,848 unique contigs with a total of 7,436,294 sequences after removing chimeras and non-fungal lineages. There were only 1246 OTUs out of 11,691 with > 10 counts, representing a total of 7,279,077 contigs across all samples. Of these OTUs, the majority (80%) fell under Ascomycota while 18% remained unclassified. These OTUs classified as fungi represented 159 species, 206 genera, 142 families, 62 orders and 20 classes. However, of the 260,014 contigs without specific fungal classification, only 2% belonged to unclassified fungal lineage based on BLAST hits against NCBI database while a majority (96%) belonged to non-fungal lineages and 2% had no taxonomic assignments.

**Figure 1:**
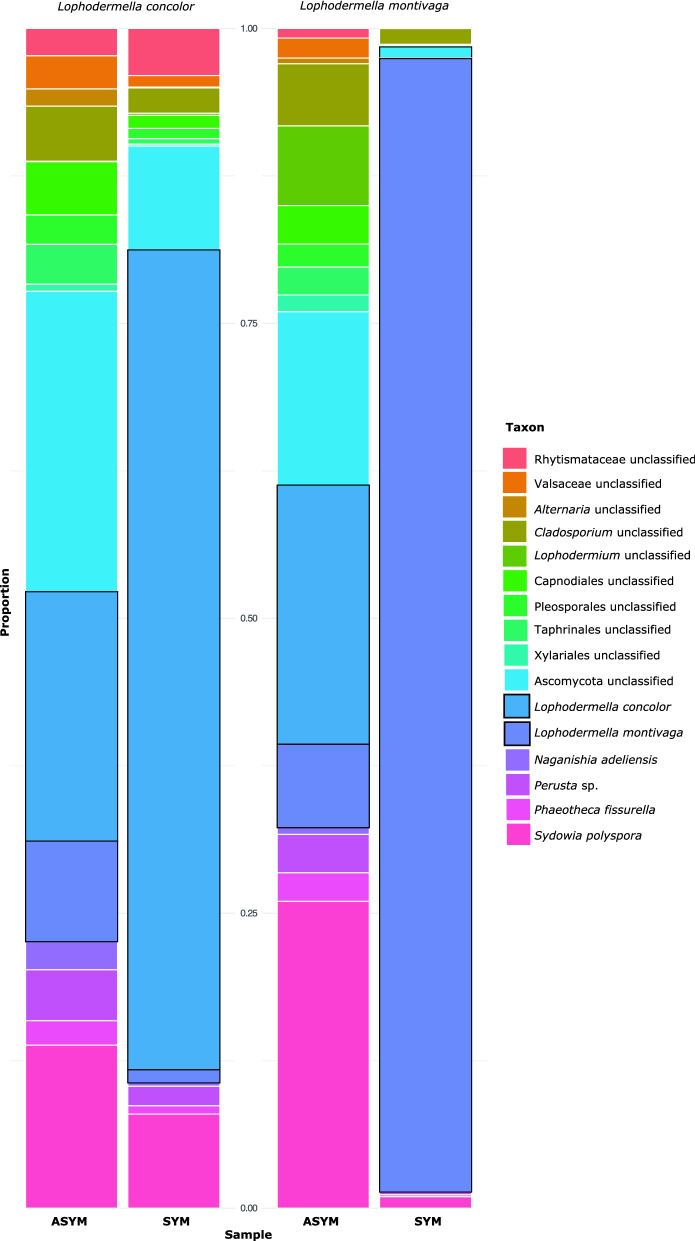
Relative abundance of fungal taxa (removing OTUs assigned as unclassified fungi) within the mycobiome across *Pinus contorta* needles that were asymptomatic (ASYM) and symptomatic (SYM) of *Lophodermella concolor* and *L. montivaga* identified through metabarcoding.

Of the fungal OTUs classified using UNITE database, *Lophodermella concolor* and *L. montivaga* dominated in their respective symptomatic needles by 67% and 96%, respectively (Fig. 1) followed by unclassified Ascomycota with an average of 5%, *Sydowia polyspora* (4%) and unclassified *Cladosporium* (2%). While one *Lophodermella* pathogen dominated in symptomatic needles (*L. concolor* in LC_SYM and *L. montivaga* in LM_SYM), a low proportion (≤ 1%) of the other *Lophodermella* species (*L. montivaga* in LC_SYM and *L. concolor* in LM_SYM) was also observed. Unclassified Ascomycota dominated among asymptomatic needles with an average of 18.1% followed by *S. polyspora* (18%) and unclassified *Cladosporium* (5%). Interestingly, *L. concolor* (19%) and *L. montivaga* (7%) were both present in asymptomatic needles albeit in low numbers relative to their symptomatic needle counterparts.

**Figure 2:**
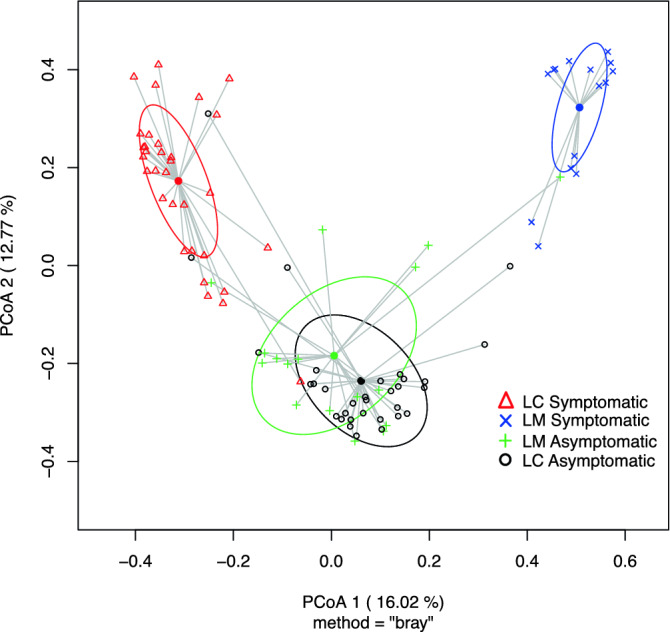
Principal coordinate analysis (PCoA) using Bray–Curtis distances based on relative abundance of fungal operational taxonomic units (OTUs) showing fungal community structure on *Pinus contorta* needles that are symptomatic or asymptomatic of *Lophodermella concolor* (LC) and *L. montivaga* (LM). Ellipses represent one standard deviation.

We found that the pathogen species and disease symptoms, accounting for site variations, were significant predictors in both alpha and beta diversity (Supplementary Table S2). A significantly higher diversity (adjusted p-values < 0.0001) was observed in asymptomatic needles compared to their symptomatic counterparts (Supplementary Table S2). While differences in diversity among asymptomatic needles was marginal (Table 1; adjusted p-value > 0.05), the diversity between needles that were symptomatic of *L. concolor* and *L. montivaga* were profoundly different (H: t-ratio = 5.799, p-value < 0.0001; 1/D: t-ratio = 4.532, p-value < 0.0001). Meanwhile, 29% of the variability was explained by pathogen and disease symptoms (Fig. 2). Despite removing contigs that matched to ‘Fungi unclassified,’ diversity remained significantly different between asymptomatic needles vs. symptomatic needles, and between needles symptomatic of *L. concolor* and *L. montivaga* (Supplementary Table S3).

**Table 1:** Diversity measures (richness, Shannon index H, inverse Simpson index 1/D) among asymptomatic and symptomatic *Pinus contorta* needles collected from Gunnison National Forest, Colorado, USA.

Needle treatment	Species	Predicted means (SE)	t-ratio	p-value
**Richness**				
Asymptomatic	*L. concolor*	66.78 (4.02)	− 0.123	0.903
	*L montivaga*	67.62 (5.57)		
Symptomatic	*L. concolor*	35.94 (4.02)	3.842	0.0002
	*L montivaga*	9.71 (5.57)		
**Shannon index (H)**				
Asymptomatic	*L. concolor*	2.13 (0.09)	− 0.157	0.876
	*L montivaga*	2.16 (0.13)		
Symptomatic	*L. concolor*	1.20 (0.09)	5.799	< 0.0001
	*L montivaga*	0.28 (0.13)		
**Inverse Simpson index (1/D)**				
Asymptomatic	*L. concolor*	0.68 (0.04)	− 0.604	0.547
	*L. montivaga*	0.72 (0.05)		
Symptomatic	*L. concolor*	0.32 (0.04)	4.352	< 0.0001
	*L. montivaga*	0.06 (0.05)

### Metatranscriptome assembly

The metatranscriptome libraries from the RNA of asymptomatic and symptomatic needle samples generated a total of 237,903,220 reads (Table 2). The assembly generated 2,079,387 transcript contigs with an average length of 552 bases. Fifty percent of the metatranscriptome sequence was covered by contigs with at least 765 bases (N50). More than 86% of the reads across all samples aligned back to the assembly (Table 2). Correlation assessment between samples within treatments and Principal Component Analysis (Supplementary Fig. S1) identified NC04-18MP sample as an outlier and thus was excluded from further analysis. The average Pearson correlation between samples within a treatment were 0.8 for LM_SYM (excluding NC04-18MP), 0.6 for LM_ASYM, 0.7 for LC_SYM and 0.7 for LC_ASYM. Notably, LC_ASYM and LM_ASYM samples were highly correlated compared to their symptomatic counterpart. The five asymptomatic and four symptomatic samples, which contained a total of 510,575 fungal OTUs, had 10,505,207 transcripts (Supplementary Table S4).

**Table 2:** Metabarcoding and metatranscriptome profile of *Pinus contorta* needle samples.

Disease symptom	Sample	DNA contigs (metabarcoding)		Raw RNA reads*	Paired reads that aligned concordantly to the assembly ≥ 1 time (%)	Overall rate (%) of alignment to the assembly
		*L. concolor*	*L. montivaga*			
Asymptomatic	CS02-18CN	1014	173	21,337,962	78.82	93.4
	TC01-19CN	2386	3706	24,537,902	79.75	93.56
Symptomatic	CS02-18CP	26,149	84	20,416,050	66.61	87.89
	SR10-19CP	22,791	0	21,485,100	71.65	91.68
Asymptomatic	LV02-18MN	22,078	18,744	22,184,468	78.33	89.96
	LV03-18MN	522	4785	25,400,132	73.79	86.48
	SR09-18MN	26,245	1422	30,543,904	77.44	94.48
Symptomatic	LV02-18MP	0	20,353	20,609,968	79.88	92.06
	NC04-18MP^†^	3	63,789	24,456,808	77.26	92.70
	SR09-18MP	0	43,121	26,930,926	78.37	92.76

Proportions (%) of raw reads that aligned to the metatranscriptome assembly and the overall alignment rate are also shown.

Cross (^†^) represents samples excluded from the analyses.

*Number of RNA reads per sample annotated as fungi through concatenated databases of NCBI-nr and JGI Mycocosm is listed on Table S4.

### Metatranscriptome differential gene expression

**Figure 3:**
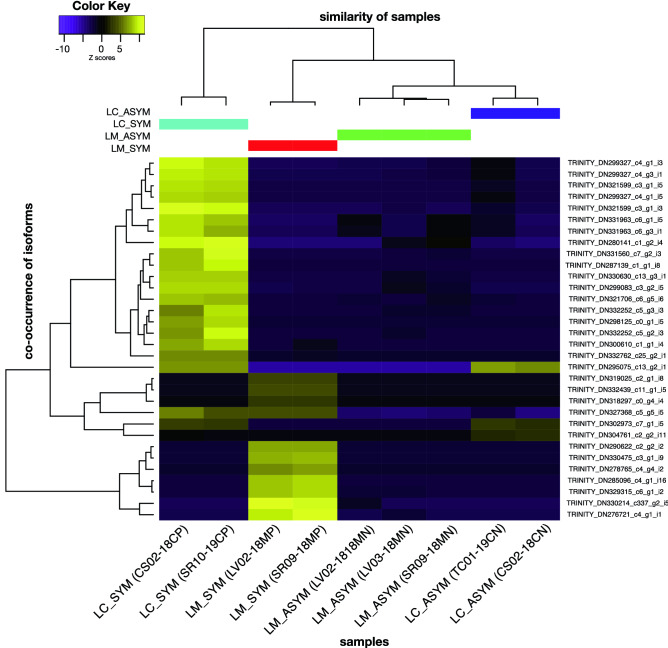
Heatmap of the differentially expressed transcripts (isoforms) in *P. contorta* needles that were asymptomatic (ASYM) and symptomatic (SYM) of *Lophodermella concolor* (LC) or *L. montivaga* (LM) based on counts per million (CPM) reads. Trinity transcripts shown are the top 10 differentially expressed features (p-value < 0.001, fold change ≥ 2) within each of the four pairwise comparisons. Similar colors at tree tips represent replicate samples within each of the following treatments: LC_ASYM (purple), LC_SYM (blue), LM_ASYM (green), and LM_SYM (red).

Differential expression profiles were similar across LC_ASYM and LM_ASYM samples (Fig. 3). In contrast, differential expression profiles between LC_SYM and LM_SYM were distinct from each other. A total of 85,798 transcripts were differentially expressed (DE) across all four comparisons: LC_ASYM_vs._LC_SYM, LM_ASYM_vs._LM_SYM, LC_ASYM_vs._LM_ASYM, and LC_SYM_vs._LM_ASYM. Collapsing identical transcripts produced a total of 51,363 transcripts with 93% (47,812) classified as non-rRNA.

The largest number of DE transcripts was observed in LC_SYM_vs._LM_SYM (38,439), followed by LM_ASYM_vs._LM_SYM (32,779) and LC_ASYM_vs._LC_SYM (14,562). Only 18 DE transcripts were found in the LC_ASYM_vs._LM_ASYM. Greater expression of transcripts was observed in symptomatic needles compared to asymptomatic ones: 14,433 (99%) in LC_SYM vs. 129 (1%) in LC_ASYM, and 32,462 (99%) in LM_SYM vs. 317 (1%) in LM_ASYM. Of the 46,895 DE transcripts in needles symptomatic of *L. concolor* and *L.montivaga*, only 1.1% were shared between species. Similarly, needles asymptomatic of *L. concolor* and *L. montivaga* shared only 0.9% of the 446 DE transcripts. These may be attributed to possible sequence divergence that separated the orthologs into distinct transcripts. Orthovenn analysis showed that symptomatic needles contained more transcripts that belong to unique protein clusters than in asymptomatic needles, with only 1% shared between them (Supplementary Fig. S2). Nearly 98% of the total protein clusters in LC_ASYM_vs._LC_SYM (2680) and LM_ASYM_vs._LM_SYM (5679) were exclusive to symptomatic needles.

**Figure 4:**
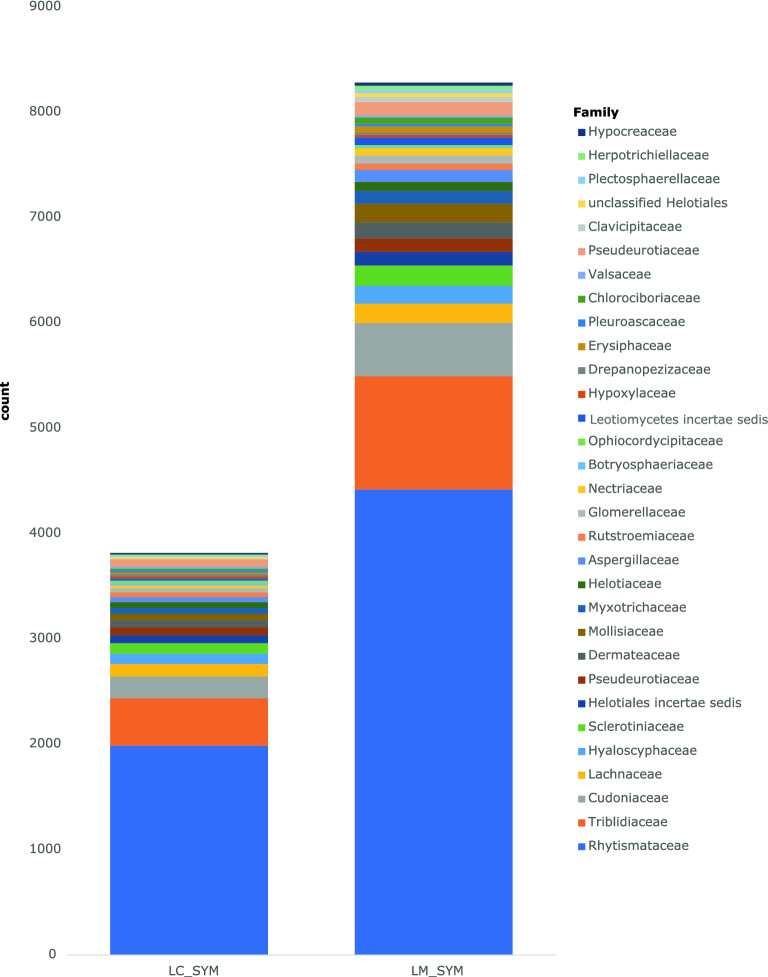
Abundance of the top 25 taxonomic families of differentially expressed (DE) fungal transcripts among *P. contorta* needles symptomatic (SYM) of *Lophodermella concolor* (LC) or *L. montivaga* (LM) when compared to their corresponding asymptomatic needles (LC_ASYM vs. LC_SYM and LM_ASYM vs. LM_SYM); in these two comparisons, no fungal DE transcripts in asymptomatic needles were among the top 25 families. Taxonomic annotations were obtained from concatenated databases of NCBI-nr and JGI Mycocosm.

Of the 85,798 DE transcripts, only 39,807 had taxonomic annotations with 61% identified as fungi. Nearly 30% and 3% of these DE transcripts were identified as bacteria and plants, respectively (Supplementary Fig. S3). The majority (98%) of the 24,289 fungal transcripts belonged to Ascomycota, followed by Basidiomycota (1.1%) and Mucoromycota (0.4%). None of the DE transcripts in LC_ASYM_vs._LM_ASYM were identified as fungi. Among the comparisons involving asymptomatic vs. symptomatic needles, no fungal taxa with > 10 DE transcripts were found in asymptomatic needles (Supplementary Table S5). However, fungal transcripts may still be present but were not significantly expressed. In symptomatic needles, many fungal DE transcripts (LC_SYM = 47% and LM_SYM = 49%) were classified under Rhytismataceae (Fig. 4). Since there are no available sequenced *Lophodermella* genomes, rhytismataceous transcripts could only be matched to other closely related genera (Supplementary Table S5).

DE plant transcripts were more abundant in symptomatic needles than their asymptomatic counterparts (Supplementary Fig. S3), with nearly twice the DE transcript count to that of asymptomatic needles. Out of the total 1317 DE plant transcripts across all four comparisons, only 31% were classified as conifers dominated by *Picea sitchensis* and were more abundant in asymptomatic needles within comparisons LC_ASYM_vs._LC_SYM and LM_ASYM_vs._LM_SYM. In contrast, transcripts classified as non-conifers were generally abundant in symptomatic needles possibly due to the lack of genome annotation for *P. contorta* host.

### Functional annotation of fungal transcripts

**Figure 5:**
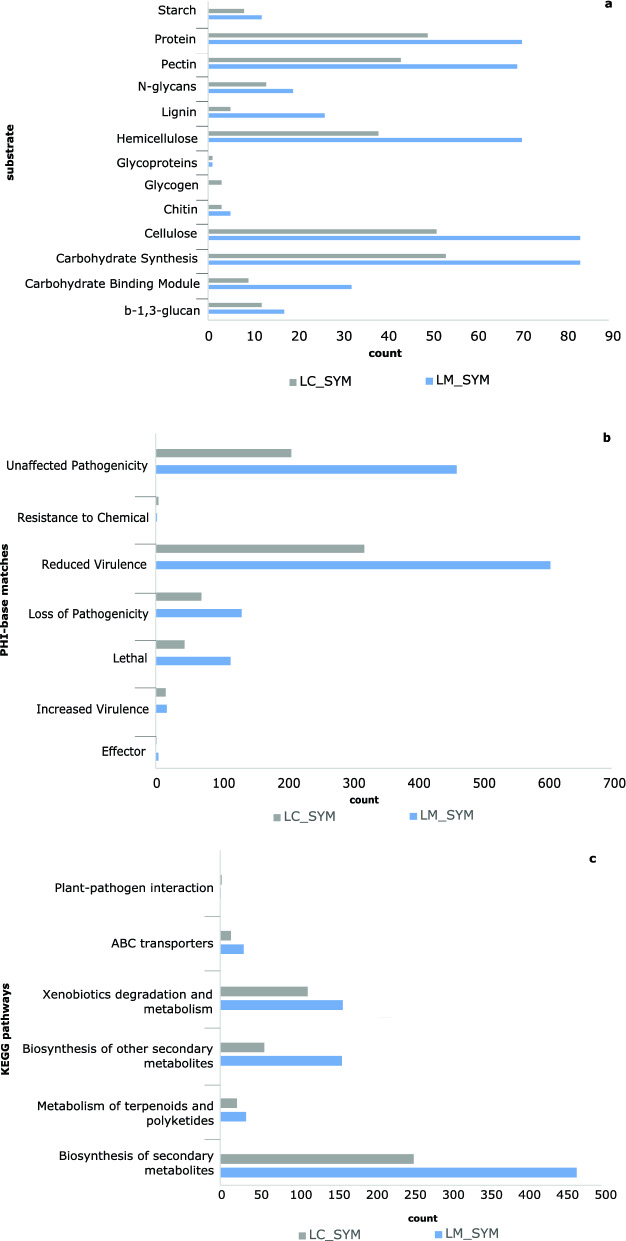
Number of differentially expressed (DE) fungal transcripts annotated as (**a**) enzymes that degrade proteins and various substrates, (**b**) genes important to pathogenicity, and (**c**) metabolic pathways in needles symptomatic of *Lophodermella concolor* (LC) and *L. montivaga* (LM) in comparisons LC_ASYM vs. LC_SYM and LM_ASYM vs. LM_SYM, respectively; in these two comparisons, no fungal DE transcripts in asymptomatic needles. Annotations were inferred from dbCAN2 and PFAM, PHI-base and KEGG, respectively.

To obtain insights into the metabolic activities of the mycobiome within healthy and diseased needles, DE fungal transcripts were compared in functional annotation databases: Pfam, PHI-base, EffectorP, dbCAN, KEGG, and Swiss-Prot and GO through Orthovenn. Roughly 80% of the total DE fungal transcripts across all three comparisons (LC_ASYM_vs.LC_SYM, LM_ASYM_vs._LM_SYM and LC_SYM_vs._LM_SYM) had annotations in at least one of the databases, and many of these were highly expressed in symptomatic needles (Fig. 5, Supplementary Fig. S4). There were no DE transcripts identified as fungi in LC_ASYM_vs._LM_ASYM. In LC_SYM_vs._LM_SYM, functions of the DE transcripts in both treatments were similar despite a low proportion of shared transcripts possibly influenced by sequence divergence between the two dominant *Lophodermella* pathogens.

The fungal community in symptomatic needles highly expressed carbohydrate-active and protein-degrading enzymes (Fig. 5a, Supplementary Table S6). Fungal proteases and peptidases were commonly found in symptomatic needles (Supplementary Table S6). While there was remarkable expression of enzymes involved in carbohydrate synthesis, other CAzymes such as glycosyl hydrolases (GH) with known functions of lignin and carbohydrate degradation were also common and highly expressed in symptomatic needles. Among comparisons of LC_ASYM_vs._LC_SYM and LM_ASYM_vs._LM_SYM, a higher expression of CAzymes, proteases and peptidases was observed in LM_SYM than in LC_SYM.

Genes related to pathogenicity were also expressed in symptomatic needles (Fig. 5b). In asymptomatic vs. symptomatic needle comparisons, more than 50% of the 604 and 1235 PHI-base hits in *L. concolor* (64%) and *L. montivaga* (59%) symptomatic needles, respectively, were associated with pathogenicity, virulence, and/or chemical resistance. Of these, only 6 and 26 *L. concolor* and *L. montivaga* transcripts, respectively, were predicted as secreted proteins. Moreover, only 1 out of 5 and 1 out of 3 EffectorP-annotated DE transcripts were predicted as secreted effectors in LC_SYM and LM_SYM, respectively, using DeepLoc. In contrast, no significant DE transcripts in asymptomatic needles had hits to EffectorP nor PHI-base databases, showing no significant pathogenic activity in asymptomatic needles.

KEGG annotations of fungal DE transcripts also showed enhanced gene expression in symptomatic needles compared to asymptomatic ones. Genes involved in the synthesis of secondary metabolites were also highly expressed in symptomatic needles (Fig. 5c). Proteins dominant in symptomatic needles were involved in the synthesis of other secondary metabolites and xenobiotics biodegradation and metabolism (Supplementary Table S7). Pathways in symptomatic needles also included membrane transport (KO02010 ABC transporters: LC_SYM = 14, LM_SYM = 31) and environmental adaptation (KO04626 Plant-pathogen interaction: LC_SYM = 2, LM_SYM = 1).

More GO annotations were detected in symptomatic needles that were related to biological processes, molecular functions and cellular components observed (Supplementary Table S8). The only two fungal DE transcripts in LM_ASYM in comparison LM_ASYM_vs._LM_SYM were related to biological processes (i.e., xanthophyll cycle and transcription elongation). Of the biological processes identified in symptomatic needles, processes such as protein and membrane transport, ubiquitin-dependent protein catabolic process, and pathogenesis were among the most common. Molecular functions common in symptomatic needles included oxidoreductase and metallopeptidase activities, and zinc and metal ion binding. Gene products or protein complexes in symptomatic needles were commonly observed in the cellular membrane.

### Functional annotation of plant transcripts

For DE transcripts taxonomically annotated as plants, functional annotation was conducted using the following protein databases: Pfam, dbCAN, KEGG, PRGdb, NCBI, and Swiss-Prot and GO in Orthovenn. Seventy-eight percent of the total 1317 plant DE transcripts across all four comparisons had annotations in at least one of the functional annotation databases, although a few (25 DE transcripts) were annotated as proteins with uncharacterized, unknown or hypothetical function.

DE plant transcripts in both asymptomatic and symptomatic needles mostly belonged to GHs and glycosyltransferase families. However, only a small proportion of transcripts (3% in LC_ASYM vs. LC_SYM and 6% in LM_ASYM vs. LM_SYM) were annotated using dbCAN2. Interestingly, albeit in low numbers, protein-degrading enzymes such as metallopeptidases and serine peptidases were found in asymptomatic needles but not in symptomatic ones.

PRGdb annotation showed higher expression of plant resistance genes in asymptomatic needles compared to symptomatic ones (Supplementary Table S9). Kinase (KIN) with transmembrane and kinase domains was the most dominant class in asymptomatic needles followed by receptor-like proteins (RLP) with TM and extra-cellular leucine-rich repeat domains. Similarly, annotated through KEGG database, abundant proteins associated with the mitogen-activated protein kinase (MAPK) signaling pathway and plant hormone signal transduction were more abundant in asymptomatic needles. Phospholipase D signaling pathway was unique to asymptomatic needles.

In contrast, the DE transcripts associated with other pathways for signal transduction were slightly more abundant in symptomatic needles (Supplementary Table S10). About 37% of the DE transcripts in needles symptomatic of *L. concolor* (47 out of 127) and *L. monitvaga* (98 out of 265) were annotated through KEGG. However, of these, only 5 were common, which included ATP citrate lyase (ACL; K01648) and translation initiation factor 5A (eIF5A; K03262) that were exclusive to symptomatic needles, and serine/threonine-protein phosphatase 2A (PP2A; K04354) and cleavage and polyadenylation specificity factor (CPSF; K14404) which were abundant in symptomatic needles. DE plant proteins in both asymptomatic and symptomatic needles were mostly related to biological processes, although GO annotated proteins were generally more abundant in symptomatic needles (Supplementary Table S11). Interestingly, in LC_SYM_vs._LM_SYM, plant proteins highly expressed in LC_SYM were related to response to stresses such as salt stress (11) and oxidative stress (4) whereas LM_SYM was mostly dominated by proteins related to pathogenesis (12) and transmembrane transport (9).

### Functional annotation of other transcripts

Despite the enrichment of eukaryotic organisms, bacterial transcripts were still recovered with a majority observed in symptomatic needles (LC_ASYM = 2 and LC_SYM = 20,687 in LC_ASYM_vs._LC_SYM, and LM_ASYM = 0 and LM_SYM = 4420 in LM_ASYM_vs._LM_SYM). Symptomatic needles contained highly abundant proteins (Supplementary Table S12) and enzymes for carbohydrate synthesis and degradation (Supplementary Fig. S5). Chitin-degrading enzymes (CE9 and GH125) were enriched, though not the most abundant, among the bacterial transcripts in symptomatic needles. GO annotations also revealed high expression of genes involved in biological processes, particularly protein transport (GO:0015031) and pathogenesis (GO:0009405).

A large proportion (54%) of the metatranscriptome remained taxonomically unclassified and the majority was identified in symptomatic needles (Supplementary Fig. S3). Of these, only 3676 out of 45,991 were functionally annotated using Pfam, PHI-base, EffectorP, dbCAN, and Swiss-Prot and GO through Orthovenn. Proteins associated with biological processes such as ubiquitin-dependent protein catabolic process and protein transport were abundant in symptomatic needles within comparisons LC_ASYM_vs._LC_SYM and LM_ASYM_vs._LM_SYM. LC_SYM was further dominated with proteins involved in pathogenesis and transcription while transposition and DNA integration were abundant in LM_SYM. While only a few, CAzymes that degrade cellulose and hemicellulose were found in symptomatic needles while only carbohydrate synthesis was observed in asymptomatic needles. Interestingly, extracellular effectors were predicted only among transcripts in symptomatic needles (LC_SYM = 5, LM_SYM = 7).

## Discussion

This study explored mycobiota shifts and changes in gene expression in the interaction among fungal endophytes, *Lophodermella* pathogens, and *P. contorta* host in the Lophodermella needle cast pathosystem. The results supported our hypothesis and revealed an adverse impact of disease on the needle mycobiota, with a significant decrease of fungal diversity as the pathogenic mycobiota dominated by *Lophodermella* pathogens becomes highly active. It further showed an elicitation of diverse plant defense mechanisms that differed in healthy and diseased needles, and between *L. concolor* and *L. montivaga* dominated mycobiota. This study also described for the first time the endophytic lifestyle of *L. concolor* and *L. montivaga*.

### Mycobiome composition and diversity

This study showed that *P. contorta* needles host a diverse community of fungal species dominated by Ascomycetes, a group commonly abundant in conifer needle tissues (e.g.^22,23^). Some fungal OTUs also remained unclassified, which highlights the diversity of potentially novel endophytes with ecological roles yet to be identified^2,24^. This study further identified that *L. concolor* and *L. montivaga* may be part of the ‘normal’ mycobiome of *P. contorta* needles as both are present in asymptomatic and symptomatic needles. The presence of pathogen in the absence of disease can occur in many pathosystems where potential pathogens are members of a healthy microbiome^25^. Since this study only examined *P. contorta* stands in Colorado, whether one or both *Lophodermella* pathogens are present in healthy needles of other *P. contorta* provenances, which have varying levels of needle cast resistance^26^, is still unknown. Alternatively, their existence in asymptomatic needles may not necessarily be part of the commensal microflora but instead are persistent infections due to their ability to evade the host immune response as endophytes^27^. Susceptible hosts that are persistently infected then act as pathogen reservoirs.

Dysbiosis in the mycobiota, which is often the result of environmental disturbances^25^, is characterized as an overgrowth of pathogens, significant depletion of other taxa, loss of beneficial microbes or a combination of these^28^. This is in contrast to a dense and diverse healthy microbiota under normal colonizing conditions^29^. We found a highly diverse fungal community in asymptomatic needles, which indicates a condition that allows many undescribed, fungal species to colonize the needle tissue. However, at the symptomatic state, diversity was significantly reduced in the needle mycobiota with significant enrichment of *Lophodermella* pathogens. This dominance may be a manifestation of *Lophodermella* pathogens outcompeting other commensal species in the mycobiota for niche and/or other resources through various strategies^30^, or a result of environmental changes favoring the growth of pathogens over other endophytes^31^.

### Fungal gene expressions in the Lophodermella needle cast pathosystem

This study showed a variety of pathogenicity-related genes (e.g., CAZymes, effectors, secondary metabolites and ABC transporters) that were highly expressed by the mycobiota, mostly Rhytismataceae, at the necrotrophic phase of the disease. These could largely be driven by *Lophodermella* pathogens as they dominate the mycobiota and colonize the needle tissue. In contrast, little to none of these genes were significantly expressed in asymptomatic needles, which could be an indication of low or absent pathogen activity. This could be part of the cryptic strategy of fungal endophytes after their initial host penetration to evade plant defense responses. Until needle senescence, latent needle pathogens exhibited either no additional growth or a slow continuous growth in intercellular spaces after initial infection^32,33^. Thus, the absence of disease in *P. contorta* could be a manifestation of *Lophodermella* pathogens evading host plant response through their marginal growth.

We found many plant cell wall degrading enzymes expressed in symptomatic needles which likely induced host necrosis and further facilitated pathogen growth. This is similar to previous observations among hemibiotrophic foliar pathogens, where cell wall degradation via glycosyl hydrolases were significantly upregulated at the latter stage of disease^34,35^. We also found effectors in symptomatic needles, which are likely necrotrophic effectors produced by hemibiotrophic or latent pathogens as they switch to the necrotrophic stage, and thus inducing host cell death^36^. This possibly allowed *Lophodermella* pathogens access to more nutrients leading to sporulation on needles^37^. Metabolic pathways were also highly active in symptomatic needles, which could indicate abundance of toxins and other metabolites at the pathogenic necrotrophic state that potentially trigger plant hypersensitive response necessary for host invasion and produce reactive oxygen species that inhibit growth of biotrophs^38^. Transport and/or secretion of these metabolites may be facilitated by ABC transporters abundant in symptomatic needles, although ABC transporters may also be involved in host penetration, survival and virulence^39,40^.

### Plant interactions in the Lophodermella needle cast pathosystem

We observed an overexpression of host defense related genes, including KINs and RLPs, in asymptomatic needles suggesting the recognition of microbial infection and thereby activating the first layer of plant inducible defense^41,42^ although RLPs could be involved in cell growth and development^43^ as part of the normal functioning of the plant host. Signaling pathways common in asymptomatic needles (e.g., MAPK and phospholipase D) could also be linked to plant hormone signaling and/or transduction with critical roles in plant growth and defense^44,45^.

Albeit rare, protein-degrading enzymes were unique in asymptomatic needles compared to symptomatic needles which could indicate plant’s response to improve tolerance against stressors. Some can be rapidly over-induced when plants are subject to unfavorable environment^46,47^ and improve plant stress tolerance^48^. We also found enzymes that could function as response to light stress resulting in photosystem repair^49^.

Plants are highly defensive and metabolically active as a response to the necrotrophic growth of hemibiotrophic pathogens^34^. We found that symptomatic needles overly expressed genes related to environmental stress, which may be triggered by the pathogenic activity of the mycobiota. Plants utilize GHs to degrade cell walls in response to stressors such as pathogen invasion and thereby inhibiting further fungal pathogen growth^50^. The presence of these plant enzymes in symptomatic needles, although rare, could be part of host defense against further growth of *Lophodermella* pathogens. Similarly, enriched proteins in symptomatic needles that were involved in programmed cell death (CPSF^51^, eIFA^52^, ACL^53^) and stress signaling (PP2A)^54^ are likely an attempt by *P. contorta* to defend itself against the pathogenic activity of the mycobiota. However, this plant defensive environment may only have eventually increased susceptibility and plant necrosis^55^, and fungal pathogen growth and development^56^.

Despite highly similar functions of genes expressed by *L. concolor* and *L. montivaga* at the symptomatic phase, gene expression by the plant differed between *L. concolor* and *L. montivaga* symptomatic needles. This could indicate unique and possibly uncharacterized strategies by each *Lophodermella* pathogen that elicit different responses in *P. contorta*, which could influence important features (e.g., morphology and host specificity) unique to each species. Similar observations were noted in two *Phytophthora* pathogens of *Theobroma cacao* where the differences in genome structure and *in planta* transcriptome expression profiles presumably resulted in differences in host range^57^.

### Characterization of other transcripts

The high expression of non-fungal genes associated with protein and substrate breakdown detected in symptomatic needles could indicate a complex interaction among endophytes, the pathogen and the host, thus further investigation needs to be conducted. Interestingly, we found an overexpression of chitin-degrading enzymes at the symptomatic phase which could have likely reduced pathogen activity. Future studies should explore the potential of these beneficial bacterial communities as a strategy to control conifer foliage diseases.

We found an abundance of transcripts with unknown identities and functions, showing the existing limitations and challenges in our understanding about microbial community interactions^58^. Nonetheless, there is little doubt about the role CAZymes and effectors play in the necrosis observed in symptomatic needles. However, whether these were proteins employed by *Lophodermella* during pathogenesis or by another organism in the community remains unknown. Using sequenced genomes of *L. concolor* and *L. montivaga* as references in transcriptome assembly would allow us to refine which transcripts belong to *Lophodermella* species within infected needle community. However, their inability to grow in culture media and lack of asexual reproductive structures present a challenge in genome sequencing. Therefore, either metagenomic or single cell approaches may be necessary to effectively capture and assemble their genomes^59^.

### *Lophodermella* as latent pathogens in *P. contorta* needles

This study provides evidence that *L. concolor* and *L. montivaga* are likely latent needle pathogens in *P. contorta*. Despite their significant presence, we did not detect pathogenic activities in asymptomatic needles which suggests a period of dormancy for *Lophodermella* pathogens. Until this study, there had been no documentation to suggest that endophytic species within the *Lophodermella* genus exist, possibly owing to their fastidious or likely obligate lifestyle. Previous observations pointed to *Lophodermella* species being active parasites^18,21^ and survival throughout the next season is possible if infected needles are not shed^60^.

With the presence of *Lophodermella* spp. in healthy and diseased *P. contorta* needles, what triggers the lifestyle transition of these pathogens and symptom development remains a question. Nonetheless, it has been shown that environmental factors could favor further growth and/or activity of latent pathogens. The enhanced sporulation and infection of *Lophodermella* pathogens with warm moisture^19^ could exacerbate pathogen invasion resulting in an increase in relative abundance. This pathogen excess in host tissue then leads to an intensified disease incidence or severity^25^ and demonstrates an imbalance in an otherwise balanced system of antagonism between disease players that leads to disease development^61^. Reference^5^ further postulated that a rapid change in endophytic density due to adverse biotic and abiotic stresses, results in premature needle cast. Alternatively, latency could be affected by processes involving physiological constraints and/or adaptation^62^ or a necessity to switch to necrotrophy for pathogen survival as H_2_O_2_ in plants accumulates^56^. Thus, more work is needed to understand the transitional cues involved in this pathosystem that would also incorporate environmental and anatomical factors at different life stages of the pathogens.

## Methods

### Sample collection and preparation

Second-year asymptomatic and symptomatic needles of *P. contorta* were randomly collected at breast height (approx. 1.37 m) from 60 trees infected with either *L. concolor* or *L. montivaga* from nine sites in Gunnison National Forest in June 2018 and 2019 (Supplementary Table S1). Symptomatic needles were brown or discolored with hysterothecia of either *L. concolor* or *L. montivaga*, while asymptomatic needles were green. From each tree, two to three needles from different fascicles (approx. 0.1 g in total) were pooled together as either symptomatic (n = 60) or asymptomatic (n = 60) needle samples. To remove superficial contamination, needles were washed with 0.2% Tween solution and vortexed at minimum speed for 10 min. Samples were then rinsed in 70% ethanol for one min and dried before storing in − 20 °C^63,64^.

To evaluate the effectiveness of removing contamination, a modified method from Ref.^64^ was used where three symptomatic and three asymptomatic samples were placed in distilled water and vortexed at minimum speed for 10 min. Four microliters of the rinse solution then served as template for PCR amplification. DNA was amplified using primers ITS1 and ITS4^65^ following methods by Ref.^66^. Amplification was observed in symptomatic needles but not in asymptomatic needle samples. Amplification in symptomatic needles was expected since many *L. concolor* and *L. montivaga* hysterothecia were mature by the time of collection and thus spores were likely easily dispersed in water during vortexing.

### Metabarcoding and metatranscriptome sequencing

DNA and RNA were extracted from asymptomatic and symptomatic needles combined per tree using a combination of methods by Refs.^67,68^ (Supplementary Data S1). DNA from 60 asymptomatic and 60 symptomatic needle samples were sent to the Genomics Center of the University of Minnesota, Minneapolis, Minnesota, USA for ITS sequencing. Fungal communities were determined by sequencing the ITS2 region [ITS3: (GCATCGATGAAGAACGCAGC) and ITS4: (TCCTCCGCTTATTGATATGC)] and reads were generated using Illumina MiSeq. Selected RNA samples from asymptomatic (n = 5) and symptomatic (n = 5) needle samples were sent to Novogene Corporation, Inc. for library preparation and sequencing (Table 2). Oligo (dT) beads and Ribo-Zero kit were used to enrich eukaryotic mRNA and remove rRNA, respectively. Raw reads from ITS and RNA sequencing were deposited to NCBI SRA database (BioProject ID Number PRJNA753461).

### Metabarcoding analysis

Quality of ITS reads were assessed using FastQC (v0.11.9)^69^. Samples with ≤ 581 reads (NC01-19MN and CS01-19CN) were excluded from the subsequent analyses. Reads were trimmed using Trimmomatic (v. 0.36)^70^ to retain only those with minimum length of 150 bp, and a threshold of 15 for each base starting at the 3′ end and for every 5 bases starting at the 5′ end. Further sequence processing was conducted using Mothur (v.1.40.5)^71^ following the MiSeq standard operating procedure (accessed 04/2020) as well as protocols developed by Ref.^72^. Contigs with a length $\ge$ 426 bp, and those containing ambiguous bases and homopolymers $\ge$ 8 bp were discarded. Sequences associated with chloroplast, mitochondria, archaea, and bacteria lineages were removed from the table of classified sequences. UCHIME^73^ was used to de novo identify and remove chimeric sequences. USEARCH, utilizing the dgc (distance-based greedy clustering) option, was used for clustering. Groups that were at least 97% similar were classified to belong to the same operational taxonomic unit (OTU). Sequences were assigned to their taxonomic units using Wang’s Naïve Bayes classifier with a cutoff value of 80^74^ and utilizing Mothur UNITE + INSD dataset (v.04022020)^75^ with additional *Lophodermella* ITS dataset. The statistical program R (v3.5.0), with the *vegan* package v.2.5–7^76^ and software packages *metagenomeseq* v.1.28.2^77^ and *phyloseq* v.1.30.0^78^, were used to analyze the data from Mothur. OTUs with low number of counts (≤ 10) were first removed to decrease error rate. Sequence depth and rarefaction curves were then obtained, using *vegan,* to assess whether the depth of sequences was sufficient to provide reasonable evaluation of the fungal diversity within samples. After merging OTUs that were similar at certain taxonomic ranks using the *phyloseq* package, the distribution of taxa with at least 1% proportion across treatments was assessed through bar plots.

To analyze the significance of the interaction between treatments on the alpha and beta diversity, only those samples in sites that have both *L. concolor* and *L. montivaga* were considered in the succeeding statistical analyses (Supplementary Table S1). Alpha diversity measures (Shannon and inverse Simpson indices) were generated using the *estimate_richness* function in *phyloseq*. Rarified richness was obtained through *vegan*. Linear models were fitted for richness, and Shannon and inverse Simpson diversity indices. Inverse Simpson diversity index was log-transformed to fit model assumptions. The interaction between pathogen species (*L. concolor* and *L. montivaga*) and disease symptoms (asymptomatic and symptomatic) was included as a variable alongside site as covariate. The *lm* and *Anova* functions from the *car* package^79^ were used to fit the model. The *emmeans* function from the *emmeans* package^80^ was used to perform pairwise comparisons.

To analyze differences in beta diversity, Principal Coordinates Analysis (PCoA) was performed using the *vegan* package where dissimilarity was calculated using Bray–Curtis distance. A constant was added using the^81^ method to correct for negative eigenvalues. To determine the differential endophyte community composition using relative abundances of OTUs, permutational multivariate analysis of variance (PERMANOVA) was implemented using *adonis2* function also from *vegan* similar to Ref.^82^. Marginal effect of the interaction between pathogen species and disease symptoms was assessed for potential significant impact while site was used to constrain permutations.

To investigate further the taxonomic identities of OTUs assigned as ‘Fungi unclassified,’ these contigs were BLAST against the NCBI-nt database. As 96% of ‘Fungi unclassified’, had BLAST hits to non-fungal lineages, a separate set of analyses was performed where contigs assigned as ‘Fungi unclassified’ and non-fungal lineages were removed from the table of classified sequences. Subsequent statistical analyses, including alpha and beta diversity analyses, were conducted using the newly derived data.

#### Metatranscriptome analysis

Quality of forward and reverse RNA sequences was evaluated by Novogene and using FastQC^69^. Raw Illumina reads containing adapter contamination, > 10% uncertain nucleotides, and > 50% low quality nucleotides (Qscore ≤ 5) among samples were removed by Novogene. No filtered reads across samples were further trimmed due to high quality Phred scores (> 30) during FastQC visual inspection. De novo metatranscriptome assembly was performed using the Trinity software (v2.11.0)^83^. To examine the representation of reads in the assembly, bowtie2 was used to capture and count the reads from individual samples that mapped back to the metatranscriptome assembly. TransRate^84^ was also used to examine the quality of the de novo assembly. Transcript abundance was estimated using the RNA-Seq by Expectation Maximization (RSEM v1.3.3)^85^. Correlation analyses of biological replicates between and across treatments were performed using the Pearson correlation matrix to check for outlier samples. Due to the low sum of mapped fragments (< 5e+6) and correlation value (< 0.02), NC04-18MP was removed from the subsequent analyses.

Differential expression of transcripts was analyzed using edgeR (v3.32.1)^86^. The count table of the Trinity transcript (isoform) abundance was filtered at counts per million of 1 and transcripts must be present in at least 1 replicate. Significantly differentially expressed (DE) transcripts were determined using the following parameters: 0.05 level of significance after using false discovery rate (FDR) for multiple testing, and a minimum fold change of 2. Data were normalized using trimmed mean of M values and fitted in a generalized linear model with four contrast arguments: (1) *L. concolor* asymptomatic (LC_ASYM) vs. *L. concolor* symptomatic (LC_SYM), (2) *L. montivaga* asymptomatic (LM_ASYM) vs. *L. montivaga* symptomatic (LM_SYM), (3) *L. concolor* asymptomatic (LC_ASYM) vs. *L. montivaga* asymptomatic (LM_ASYM), and (4) *L. concolor* symptomatic (LC_SYM) vs. *L. montivaga* symptomatic (LM_ASYM).

SortMeRNA was used to distinguish the non-coding regions (rRNA) among the DE transcripts^87^. The longest open reading frames of coding regions were predicted using orfipy^88^. The resulting protein products were then subject to MMseqs2 search^89^ against the following databases to predict the taxonomy, protein domains, and proteins related to pathogen-host interactions: concatenated databases of NCBI nr^90^ and published genome sequences including those of Rhytismataceae species stored at JGI Mycocosm^91^, PfamA full 2021^92^, and PHI-base 4.10^93^, respectively. Overall, only the annotated transcripts with an e-value < 1e−05 were considered. Transcripts with coverage and identity ≥ 50% in the concatenated NCBI nr and JGI Mycocosm database and PHI-base were retained. This concatenated database was used to sort fungal and plant transcripts.

Effectors (with ≥ 95% probability) and carbohydrate active enzymes (CAZymes) were predicted using EffectorP 2.0^94^ and dbCAN2^95^, respectively. Within dbCAN2, transcripts with hits in at least two of the three databases (HMMER, DIAMOND and Hotpep) were considered. Metabolic pathways of predicted proteins (FDR < 0.05) were searched through BlastKOALA^96^ using the family_eukaryotes KEGG database with the ‘fungi’ or ‘plant’ taxonomy option^97^. Orthologous gene clusters among treatments and their gene ontology (GO) annotations were determined through Orthovenn2^98^ with an e-value of < 1e−2 and inflation value of 1.5. Expressed plant receptor genes with > 50% identity were determined through PRGdb 3.0^99^. DeepLoc 1.0^100^ was used to predict secreted proteins.

## Supplementary Information

Supplementary Tables.

Supplementary Information.

## Data Availability

The raw sequence data used in the analyses are available at the Sequence Read Archive of the National Center for Biotechnology Information (BioProject ID Number PRJNA753461).
