# Supplementary material for: Transitions of foliar mycobiota community and transcriptome in response to pathogenic conifer needle interactions
Source: Sci Rep. 2022 May 12;12:7832. doi: 10.1038/s41598-022-11907-0 (PMC9098639; doi:10.1038/s41598-022-11907-0)
Supplement: Supplementary file 2 — Supplementary Information. [file 41598_2022_11907_MOESM2_ESM.pdf]

Article Title: Community and transcriptome foliar mycobiota transitions in response to pathogenic conifer needle interactions

Authors: Jessa P. Ata, Jorge R. Ibarra Caballero, Zaid Abdo, Stephen J. Mondo and Jane E. Stewart

Supplementary Figure S1. Heatmap of the isoform correlation (A) and Principal Component Analysis (B) between *Pinus contorta* needle samples that were asymptomatic (ASYM) and symptomatic (SYM) of *Lophodermella concolor* (LC) and *L. montivaga* (LM). Similar colors at tree tips represent replicate samples within each of the following treatments: LC_ASYM (purple), LC_SYM (blue), LM_ASYM (green), and LM_SYM (red).

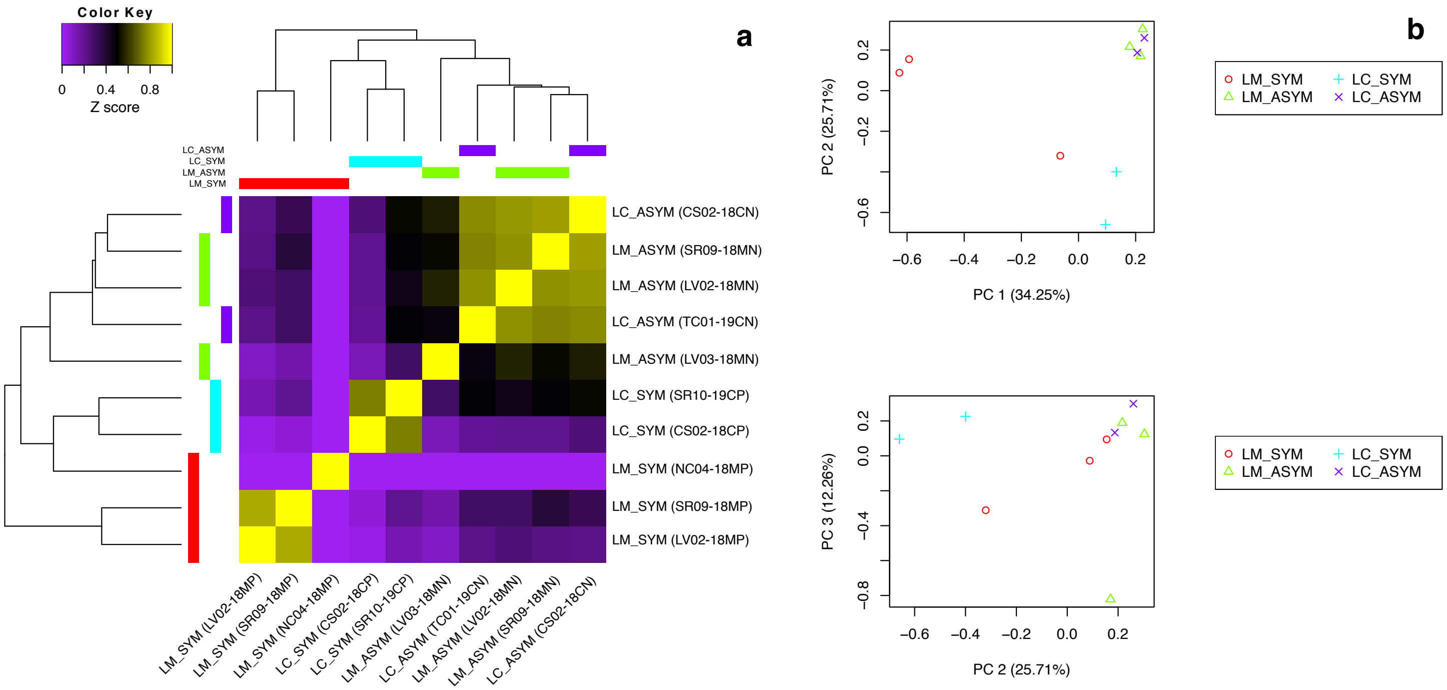

Supplementary Figure S2. Number of shared and unique orthologous protein clusters between (a) *Pinus contorta* needles symptomatic and asymptomatic of *Lophodermella concolor* (LC_ASYM vs. LC_SYM) and (b) *L. montivaga* (LM_ASYM vs. LM_SYM), and (c) between symptomatic needles of *L. concolor* and *L. montivaga* (LC_SYM vs. LM_SYM) inferred from Orthovenn2. Bold numbers correspond to the number of protein clusters.

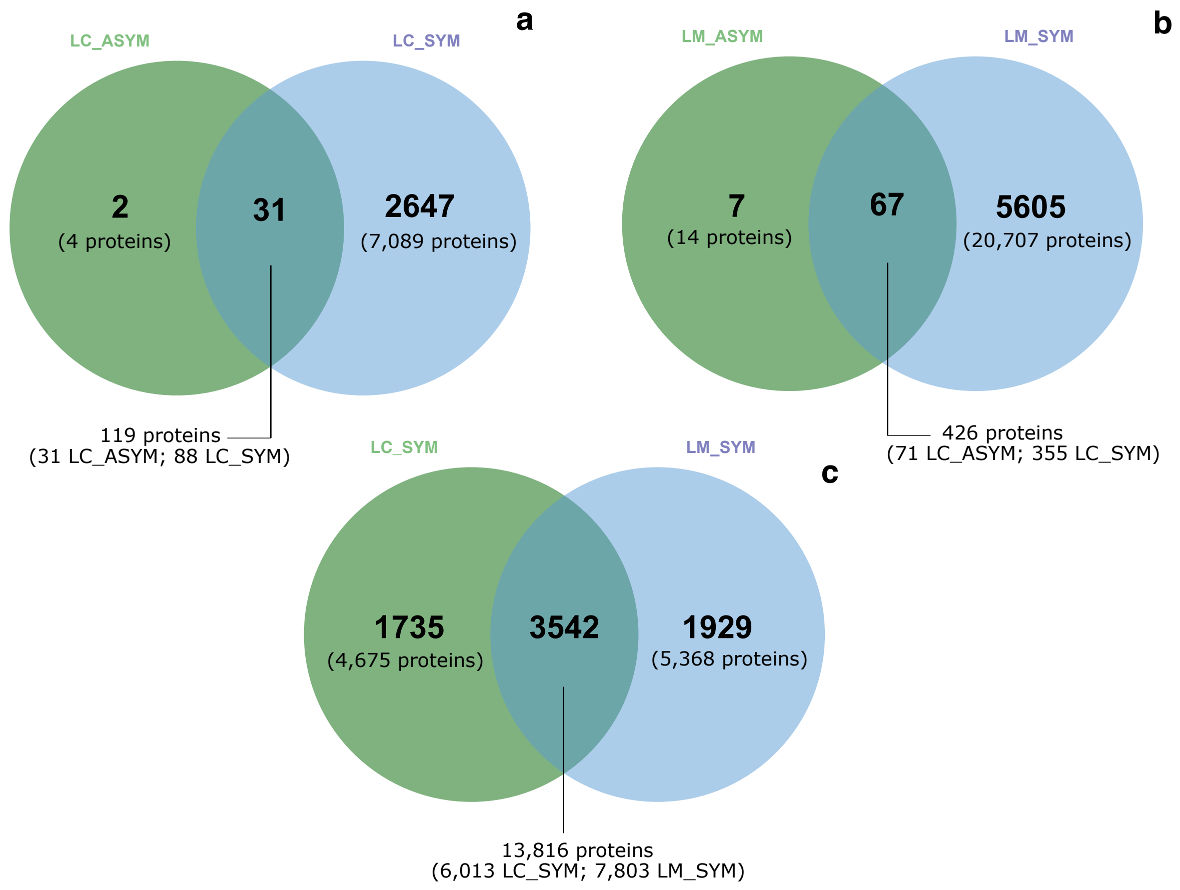

Supplementary Figure S3. Taxonomic lineage (a) and fungal phyla (b) of the significantly differentially expressed transcripts (FDR < 0.05, p-value < 0.05) across all comparisons determined through mmseqs2 search in the concatenated databases of NCBI-nr and JGI Mycocosm. Except for LM_ASYM with 2 DE transcripts in comparison LM_ASYM vs. LM_SYM, no fungal taxa were observed among asymptomatic needles across other comparisons.

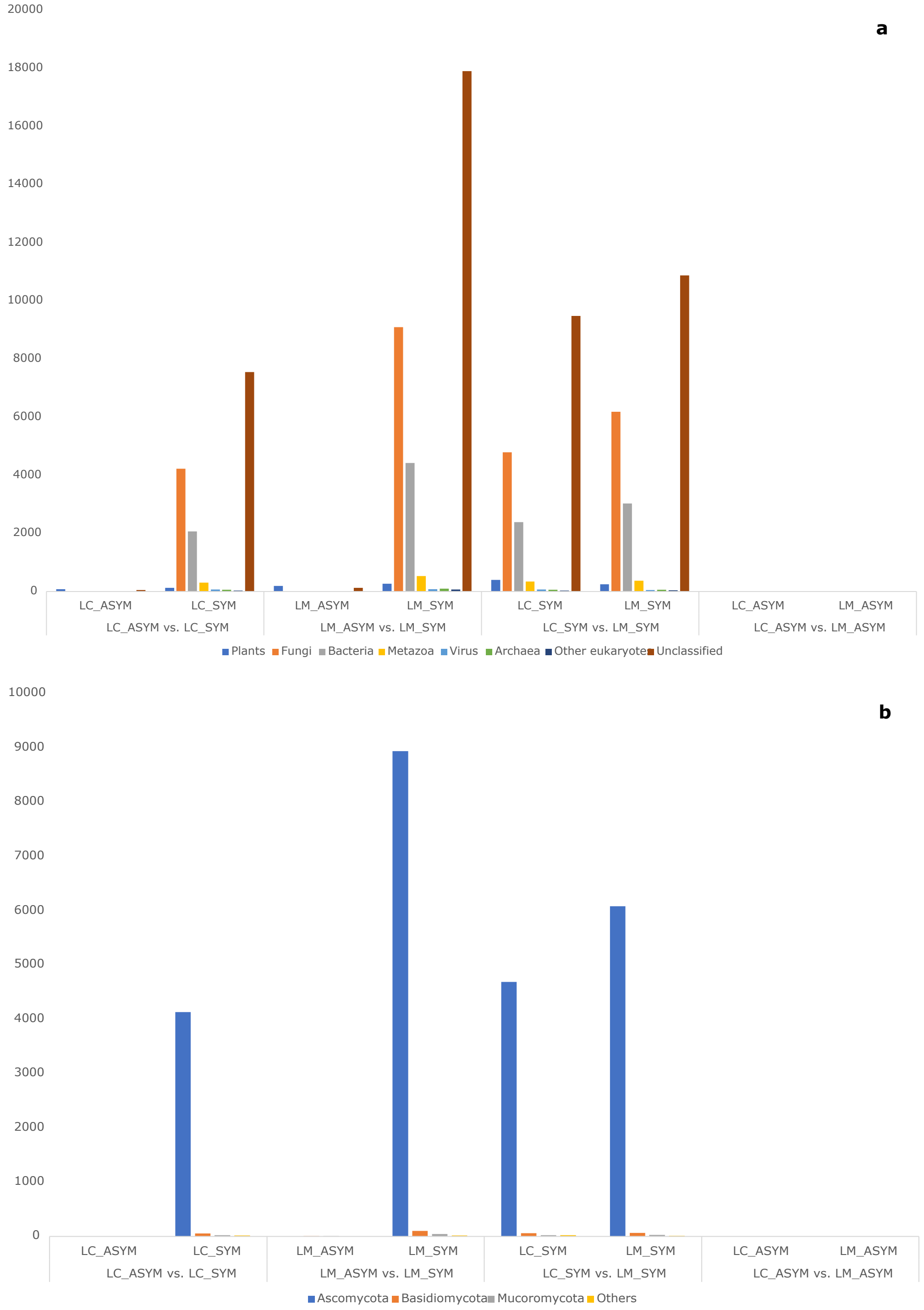

Supplementary Figure S4. Number of differentially expressed (DE) fungal transcripts annotated as (a) enzymes that degrade proteins and various substrates, (b) genes important to pathogenicity, and (c) metabolic pathways in needles symptomatic of *Lophodermella concolor* (LC) and *L. montivaga* (LM) in comparison LC_SYM vs. LM_SYM. Annotations were inferred from dbCAN2 and PFAM, PHI-base and KEGG, respectively.

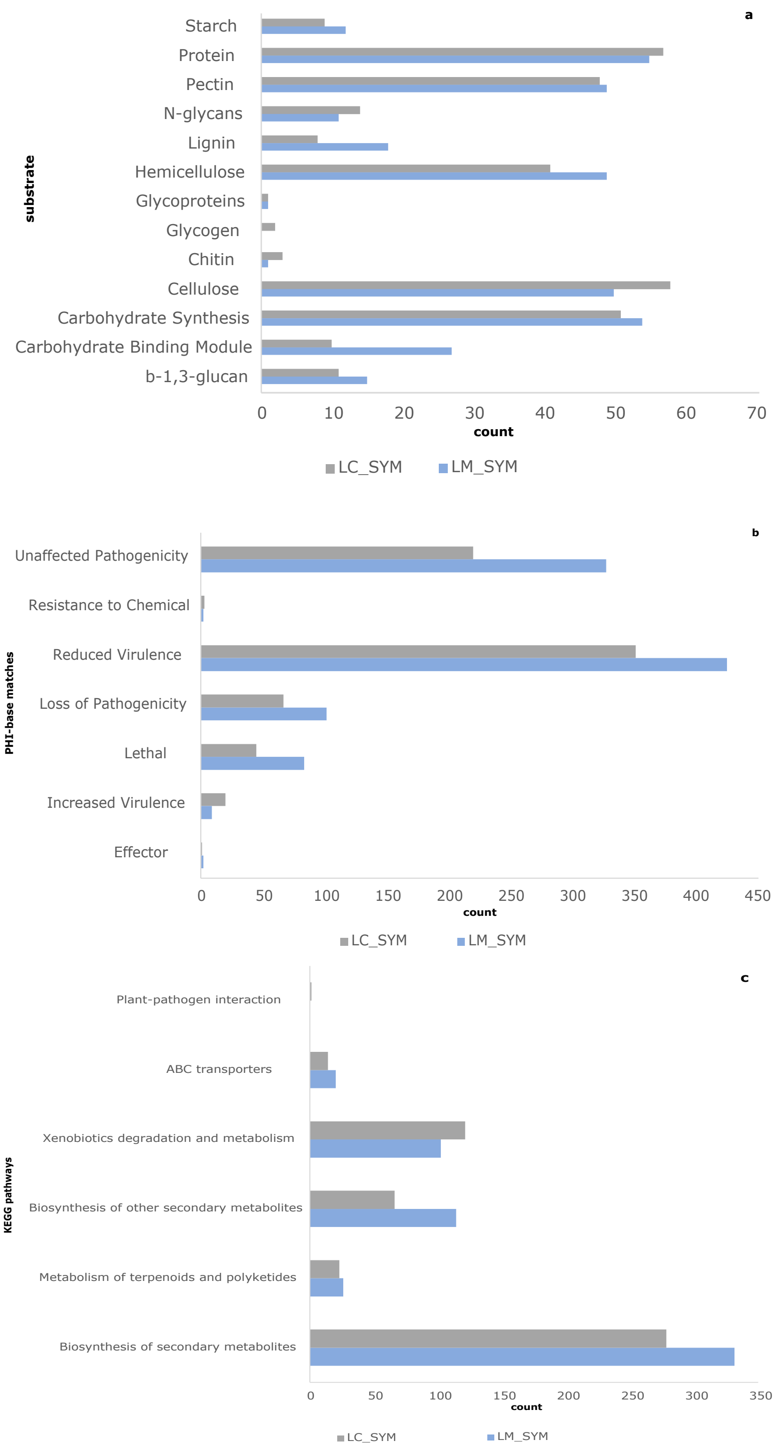

Supplementary Figure S5. Number of bacterial enzymes that degrade various substrates that were differentially expressed between (a) *Pinus contorta* needles symptomatic and asymptomatic of *Lophodermella concolor* (LC_ASYM vs. LC_SYM) and *L. montivaga* (LM_ASYM vs. LM_SYM) and (b) between symptomatic needles of *L. concolor* and *L. montivaga* (LC_SYM vs. LM_SYM) inferred from dbCAN2.

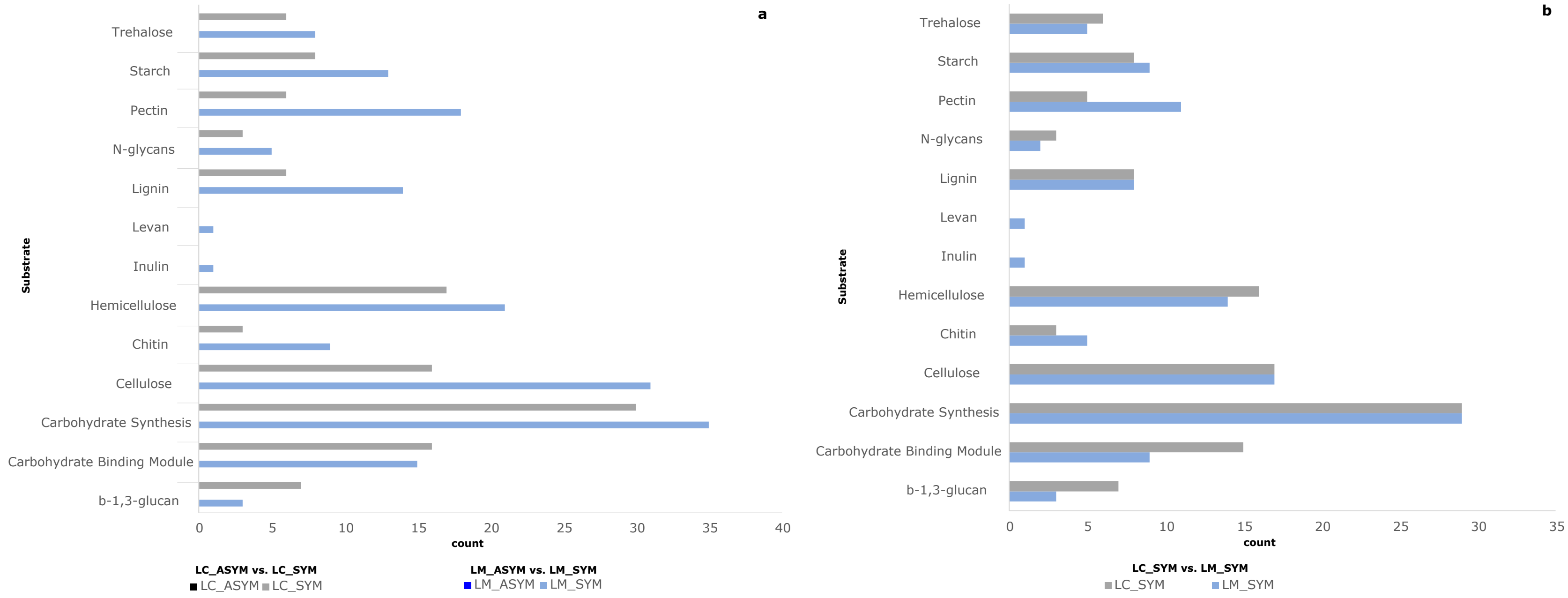

Supplementary Data S1 Genomic DNA and RNA extraction protocol to extract DNA and RNA of Pinus contorta needles from Colorado, USA that were asymptomatic and symptomatic of Lophodermella concolor and L. montivaga. Volumes were adjusted for small amount of sample.

DAY 1

1. In 2mL tubes, add clean metal and glass beads with the samples. Grind samples in FastPrep after submerging in liquid nitrogen. Repeat until samples are in powder form. Samples must be stored in -80°C prior to grinding.
2. Add 500μL of warm extraction buffer and 10μL dithiothreitol (DTT) to the samples. Run in FastPrep once then incubate the samples for 20mins at 65°C.
3. Add equal volume of chloroform:isoamyl alcohol 24:1, and mix with the extraction buffer by inversion. Centrifuge the samples to 10,000g for 15mins and transfer the aqueous phase (~400μL) into new tubes.
4. Add a quarter volume (~100μL) of 10 M lithium chloride (LiCl) and mix the solution by inversion. Precipitate the total RNA overnight at 4°C.

DAY 2

5. Centrifuge samples at 10,000g for 30mins at 4°C. Pour out supernatant (gDNA) and transfer to new 1.5mL tubes. Leave the pellet to dry (totRNA).

Genomic DNA extraction

6. Add 3 M NaAc (1/10 volume of supernatant) and isopropanol (1 volume of supernatant) to the supernatant (gDNA from step 5). Mix the solution by inversion and incubate for 5mins at room temperature. Centrifuge the samples at 10,000g for 30mins at 4°C.
7. Remove supernatant in the gDNA sample and resuspend pellet in 350μL of 1.2 M NaCl. Add 2μL of RNAse A to the samples and incubate for 30mins at 37°C.
8. Add 1 volume of chloroform:isoamyl alcohol 24:1 and mix the solution well. Centrifuge for 5mins at 10,000g and transfer the upper phase of the solution to new tubes.
9. Add 0.6 volume of ice-cold isopropanol and mix well. Incubate the sample at -20°C for at least 15 mins (or overnight).
10. Centrifuge the sample for 20mins at 13,000g at 4°C. Decant supernatant and add 1mL of 70% cold ethanol.
11. Centrifuge the samples for 3mins at 13,000g at 4°C. Carefully decant the solution using pipette avoiding pellet. Air dry the pellet.
12. Resuspend pellet in TE buffer or molecular grade water. Store DNA sample in -20°C.

Total RNA extraction

13. Dissolve the pellet (from step 5, totRNA) in 400μL of SSTE buffer (preheated at 65°C). Add same volume of chloroform:isoamyl alcohol 24:1 and mix well. Centrifuge for 15mins at 10,000g.
14. Transfer aqueous phase in 2mL tubes. Add 100% ethanol (three times the volume of the aqueous phase) and mix well. Precipitate overnight at -80°C.

DAY3

15. Centrifuge sample from step 13 at 10,000g at 4°C for 30mins. Pipette out the supernatant and air dry the pellet. Dissolve the pellet in molecular grade or nuclease free water. Store in -80°C.
